# Encapsulation of lime peel extract in alginate–gelatin microbeads and its potential for browning inhibition in *Musa* spp. tissue culture

**DOI:** 10.1038/s41598-026-57037-9

**Published:** 2026-06-11

**Authors:** Nandang Permadi, Pramesthi Reitza Navisya Vasall, Farah Aprisza Sheelmarevaa, Tatang Wahyudi, Winda Puspitasari, Mohamad Nurzaman, Febri Doni, Euis Julaeha

**Affiliations:** 1 Doctorate Program in Biotechnology, Graduate School, Universitas Padjadjaran, Bandung, 40132 West Java Indonesia; 2 Research Center for Food Crops, Research Organization for Agriculture and Food, National Research and Innovation Agency, Cibinong, 16911 West Java Indonesia; 3 Department of Chemistry, Faculty of Mathematics and Natural Sciences, Universitas Padjadjaran, Jatinangor, 45363 West Java Indonesia; 4 Research Center for Advanced Material, Research Organization for Nanotechnology and Material, National Research and Innovation Agency, Tangerang Selatan, 15310 Banten Indonesia; 5 Department of Biology, Faculty of Mathematics and Natural Sciences, Universitas Padjadjaran, Jatinangor, 45363 West Java Indonesia

**Keywords:** Antioxidant, *Citrus aurantiifolia* peel extract, microbeads, explant browning, *Musa* spp. tissue culture, Biochemistry, Biological techniques, Biotechnology, Plant sciences

## Abstract

Tissue culture micropropagation is the most effective method for propagating *Musa* spp. explants; however, enzymatic browning poses a major obstacle. Lime peel extract (LPE) has demonstrated antioxidant activity against browning in *Musa* spp. cultures, though its effectiveness is limited by instability and rapid release. Encapsulation technology using sodium alginate and gelatin biopolymers addresses these limitations by protecting active compounds, enabling controlled release, and extending shelf life. In this study, LPE was encapsulated with a yield of 79.5%, encapsulation efficiency (EE) of 80.93%, and extract content (EC) of 24.2%. The resulting microbeads were approximately spherical, although their surface is slightly irregular, averaging 1.2 μm in size, and exhibited release kinetics consistent with the Korsmeyer-Peppas model, confirming delayed LPE release. The microbeads showed thermal stability up to 100 °C, maintaining 23.26% EC and 77.8% EE over three weeks. LPE microbeads were more effective in reducing the browning index in Kepok Tanjung and Barangan explants compared to unencapsulated LPE. These findings indicate that LPE, particularly in its encapsulated form, can effectively suppress browning in *Musa* spp. tissue cultures.

## Introduction

Plantains and bananas (*Musa* spp.) are staple food crops in many developing countries, providing both essential nutrition and a source of income^1–3^. At present, the most effective method for producing *Musa* spp. explants relies on plant tissue culture technologies^4,5^. This approach enables the production of disease-free and high-quality explants, generating uniform plants in a relatively short time while supporting vigorous growth in subsequent cycles^6,7^. However, one of the major obstacles in the micropropagation of *Musa* spp. via tissue culture is browning^7,8^.

Another critical challenge is explant browning, which arises from various chemical reactions, including caramelization, phenylalanine deamination, carbonyl–amine interactions, secondary *o*-quinone reactions, lipid oxidation, and enzymatic oxidation^9,10^. Among these, enzymatic browning is the primary cause of tissue discoloration in in vitro plant cultures^11^. This process leads to detrimental changes in explant tissue, often resulting in death and failure of regeneration^12^, thereby reducing explant survival and competitiveness. Numerous studies have investigated strategies to suppress browning in banana tissue cultures^13–15^. Generally, these strategies involve removing phenolic compounds, modifying redox potential, inhibiting phenol oxidase activation, or reducing phenolase activity and substrate availability through treatments such as immersing explants in anti-browning solutions, supplementing the culture medium with inhibitors, or manipulating culture conditions^14–16^. Given these limitations, there is a need for alternative natural antioxidants to control browning.

*Citrus aurantiifolia* (Christm.) Swingle, commonly known as key lime, is widely cultivated across Asia and traditionally used to treat cough, reduce phlegm, and alleviate influenza and acne^17–19^. Its peel is rich in diverse phytochemicals, including polyphenols, flavonoids, saponins, steroids, and terpenoids^20–22^. Lime peel extract (LPE) has demonstrated multiple bioactivities, such as anticancer, antioxidant, antidiabetic, antibacterial, anti-inflammatory, antiviral, and insecticidal effects^23–25^. In particular, previous studies have confirmed its strong antioxidant activity^26^.

Despite these properties, the practical use of plant-derived extracts is often limited by their instability to oxygen, moisture, and storage conditions, as well as drawbacks such as rapid release, poor solubility, and low bioavailability^27,28^. To overcome these challenges, encapsulation technology has been widely applied. Encapsulation protects bioactive compounds from environmental degradation, preserves their functional and organoleptic properties, enables controlled release, and extends product shelf life^29^. Previous research has shown that LPE can be successfully encapsulated into microbeads with desirable physicochemical properties^30^. This process commonly employs the external ionotropic gelation method, which is favored due to its simplicity, low cost, absence of organic solvents, and mild preparation conditions that avoid toxicity and yield environmentally friendly microbeads^31^. Typically, this approach uses alginate–gelatin coatings and calcium chloride (CaCl₂) as cross-linking agents.

To date, no study has evaluated the use of encapsulated LPE as an antioxidant agent to mitigate browning in *Musa* spp. tissue cultures. Therefore, this research aims to apply LPE microbeads in culture media and assess their effectiveness in reducing explant browning during in vitro cultivation.

## **Materials and methods**

### Materials

*C. aurantiifolia* was obtained from plantations in Curah Jati Village, Purwoharjo District, Banyuwangi Regency, East Java, Indonesia. The specimen of *C. aurantiifolia* was verified and documented at the Herbarium Jatinangoriense, Department of Biology, Faculty of Mathematics and Natural Sciences, Universitas Padjadjaran, under the collection reference number 45/HB/02/2021, and identified by Joko Kusmoro. Meanwhile, the *Musa* spp. explants used consisted of one banana cultivar, Barangan (*M. acuminata*), and one plantain cultivar, Kepok Tanjung (*M. paradisiaca*), which were obtained from the Indonesian Tropical Fruits Research Institute (IP2TP), Ministry of Agriculture, Subang, West Java, Indonesia.

### LPE extraction

LPE was obtained using a maceration method following the procedure previously described by Nurzaman et al.^5^. The peel of *C. aurantiifolia* was cleaned, dried at room temperature, and ground into a fine powder (simplicia). The powder was soaked in ethanol until fully submerged and left to stand overnight. The filtrate was then collected, and the residue was re-soaked in ethanol. This process was repeated for up to 72 h (3 × 24 h). The extract was concentrated using a Büchi R-200 rotary evaporator at 50 °C.

### LPE microbeads preparation

The LPE microbeads were prepared using the external ionotropic gelation method following the procedure of Julaeha et al.^30^. The beads were formed through the reaction of sodium alginate and gelatin as coating polymers, LPE as the encapsulated material, and calcium chloride as the cross-linking agent. Each polymer (sodium alginate and gelatin) was dissolved in 50 mL of distilled water. A sodium alginate solution at a concentration of 1.75% (w/v) was mixed into a 0.25% (w/v) gelatin solution and homogenized for 5 min at 600 rpm. Subsequently, 300 mg of LPE was added to the polymer solution and stirred at 1,250 rpm until fully dissolved.

The resulting dispersion was dropped into 100 mL of 2% (w/v) calcium chloride solution using a 23-G syringe needle while stirring at 250 rpm. The dripping height was adjusted to 16 cm. The formed microbeads were allowed to stabilize under continuous stirring for 60 min, then filtered. The obtained LPE microbeads were washed with distilled water and dried at room temperature. The yield of the LPE microbeads was determined using the following equation:

$$\:\mathrm{Y}\mathrm{i}\mathrm{e}\mathrm{l}\mathrm{d}\:\left(\%\right)=\frac{{\mathrm{W}}_{2}\:\left(\mathrm{g}\right)}{{\mathrm{W}}_{1}\:\left(\mathrm{g}\right)}\:\times\:100\%$$

Where W_1_ = initial mass used (g) and W_2_ = mass obtained (g).

### Characterization of LPE microbeads

#### Measurement of extract content and encapsulation efficiency

The extract content (EC) and encapsulation efficiency (EE) were determined using a Perkin Elmer Lambda 35 UV–vis spectrophotometer. For analysis, 0.1 g of LPE microbeads was finely ground and dissolved in 10 mL of solvent in a volumetric flask. The absorbance of the resulting solution was recorded and quantified against a calibration curve prepared from LPE standards. EC and EE values were then calculated according to the equations previously described by Julaeha et al. (2023):

$$\:\mathrm{E}\mathrm{C}\:\left(\%\right)=\frac{{\mathrm{W}}_{1}\:\left(\mathrm{g}\right)}{{\mathrm{W}}\:\left(\mathrm{g}\right)}\:\times\:100\%$$

$$\:\mathrm{E}\mathrm{E}\:\left(\%\right)=\frac{{\mathrm{W}}_{1}\:\left(\mathrm{g}\right)}{\mathrm{W}_{2}\:\left(\mathrm{g}\right)}\:\times\:100\%$$

Where W = weight of microbeads; W_1_ = amount of extract coated; and W_2_ = weight of LPE used.

#### Particle size analysis

Particle size was measured using a Beckman Coulter LS 13 320 particle size analyzer (PSA), equipped with an optical Fraunhofer system and operated with water as the dispersing medium.

#### Morphological characterization

The morphology of the microbeads was examined with a JEOL JSM-6510 scanning electron microscope (SEM) operated at 10 kV, using a backscattered electron imaging (BEI) detector.

#### Fourier transform infrared spectroscopy (FTIR)

FTIR spectra were recorded with a Perkin Elmer Spectrum 100 spectrometer in the range of 450–4,000 cm⁻¹, employing KBr disk preparation.

#### Thermal stability analysis

Thermal behavior of the microbeads was assessed using a NETZSCH TG 209 F1 Libra thermogravimetric analyzer (TGA). Measurements were carried out from 26 to 1,000 °C at a heating rate of 20 °C/min under a nitrogen atmosphere.

### The kinetics of LPE release from microbeads

The release percentage of LPE from the microbeads was further evaluated to determine the amount of extract liberated over time. A total of 0.1 g of microbeads was finely ground and dispersed in 10 mL of ethanol. The released LPE was quantified daily for seven consecutive days using a UV–Vis spectrophotometer, and the absorbance values were interpolated against an LPE calibration curve. After measurement, the aliquot was returned to the original solution to maintain sink conditions. A similar procedure was conducted under two temperature conditions, 27 °C and 121 °C, to compare release profiles.

The release kinetics of LPE from the microbeads was analyzed using various mathematical models: the zero-order model (describing the cumulative percentage of LPE released over time), the first-order model (logarithmic relationship between the percentage of unreleased LPE and time), the Higuchi model (release percentage relative to the square root of time), the Hixson–Crowell model (cubic root of the remaining LPE versus time), the Korsmeyer–Peppas model (logarithmic correlation of release percentage and time), and the Avrami model (characterizing non-linear release kinetics influenced by structural transitions). The mathematical expressions of these models are as follows:

Zero-order: *M*_*t*_ */ M*_*∞*_ = *kt*.

First-order: ln (1 –*M*_*t*_ */ M*_*∞*_) = *-kt*.

Higuchi model: *M*_*t*_ */ M*_*∞*_ = *kt*^1/2^

Hixson-Crowell model: *M*_*t*_^1/3^ – *M*_*∞*_ ^1/3^ = *kt*.

Korsmeyer-Peppas model: *M*_*t*_ */ M*_*∞*_ = *kt*^*n*^.

Avrami model: ln(–ln *R*) = *n* ln(t) + *n* ln *K*(*t*).

In those equations, *t* represents the release time (minutes), *M*_*t*_ */ M*_*∞*_ denotes the fraction of LPE released at time *t*, *k* is the release rate constant, and *n* is the release exponent that explains the mechanism of release. The most appropriate kinetic model was identified based on the correlation coefficient (R²), where values approaching 1 indicate the best fit^32–34^.

### Microbeads storage stability

The storage stability of LPE microbeads was assessed following the method of Pratiwi et al.^33^. A 0.1 g portion of LPE microbeads was suspended and kept at room temperature for three weeks. The amount of extract released into the suspension was measured weekly using a UV–Vis spectrophotometer, after which the EE and EC were determined.

### Treatment and maintenance of explants

The LPE with the highest antioxidant activity, as reported by Permadi et al.^26^, was selected as the treatment reference and applied at three concentration levels: IC₅₀, 2×IC₅₀, and 3×IC₅₀, corresponding to 0.66, 1.32, and 1.98 mg/mL, respectively. For treatments using LPE microbeads, concentrations were adjusted based on the EC value to match IC₅₀, 2×IC₅₀, and 3×IC₅₀, resulting in equivalent microbead concentrations of 2.73, 5.46, and 8.19 mg/mL. Each concentration of LPE and microbeads was incorporated into Murashige and Skoog (MS) medium supplemented with 2.5 ppm benzylaminopurine (BAP) and 0.1 ppm thidiazuron (TDZ). Explants of *M. paradisiaca* var. Kepok Tanjung and *M. acuminata* cv. Barangan were then cultured under a light intensity of 1,000–4,000 lx, at 25 ± 2 °C and 80% relative humidity, for 21 days during the shoot initiation phase within a one-month period.

### Browning index measurement

The effect of treatments on browning in Kepok Tanjung and Barangan tissue cultures was assessed by calculating browning index. Visual observation of browning was conducted on the 21st day after planting (DAP). The browning index was calculated using the following equation:

$$\text{Browning Index} \left(\%\right)=\frac{\text{Number of browned explants} \times \text{browning level}}{\text{Total explants} \times \text{highest browning level}}\times 100\%$$

The browning level of explants was categorized as follows Wu et al.^10^:

Level 0 — no browning;

Level 1 — browning occurs only on the cut surface of the explant;

Level 2 — browning appears in the culture medium with an area less than 0.5 cm², with the explant at the center;

Level 3 — browning appears in the culture medium with an area greater than 0.5 cm², with the explant at the center.

### Statistical analysis

The statistical analysis was conducted using Version 30.0.0.0 of the Statistical Package for Social Sciences (SPSS) (SPSS Inc., Chicago, USA). Employing Duncan’s multiple range test and an analysis of variance (ANOVA) at a 95% confidence level (*p* ≤ 0.05), significant differences among the samples were discerned.

## Results and discussion

### LPE microbeads

Microencapsulation of LPE using the external ionotropic gelation method with alginate–gelatin matrices yielded an output of 79.50 ± 0.03%, with an EC of 24.20 ± 1.04% and an EE of 80.93 ± 1.94%. The yield and EE values were consistent with previous studies that also utilized alginate–gelatin polymer combinations^30^. In contrast, the EC value obtained was higher than that reported in earlier work, although it remains relatively low. Therefore, further investigation is required to significantly enhance EC without compromising yield and EE.

The combination of alginate and gelatin polymers offers advantages in reducing bead porosity, thereby improving encapsulation efficiency. The interaction between the carboxyl groups of sodium alginate and the amino groups of gelatin facilitates the formation of ionic and hydrogen bonds, leading to the generation of amide groups. The addition of CaCl₂ as a cross-linking agent was chosen due to its physiological non-toxicity and ability to readily form hydrogels through interactions with negatively charged polymers. The alginate–Ca²⁺ matrix is also known to improve the mechanical strength of microbeads^30^. During gel formation, two guluronate residues of alginate create cavities that serve as binding sites for Ca²⁺ ions. This interaction, described as the “egg-box” model, produces a three-dimensional gel network. The process involves the sequential formation of monocomplexes, egg-box dimers, and ultimately multimers that are fully cross-linked with Ca²⁺ ions^34^.

### Characterization of LPE microbeads

**Fig. 1:**
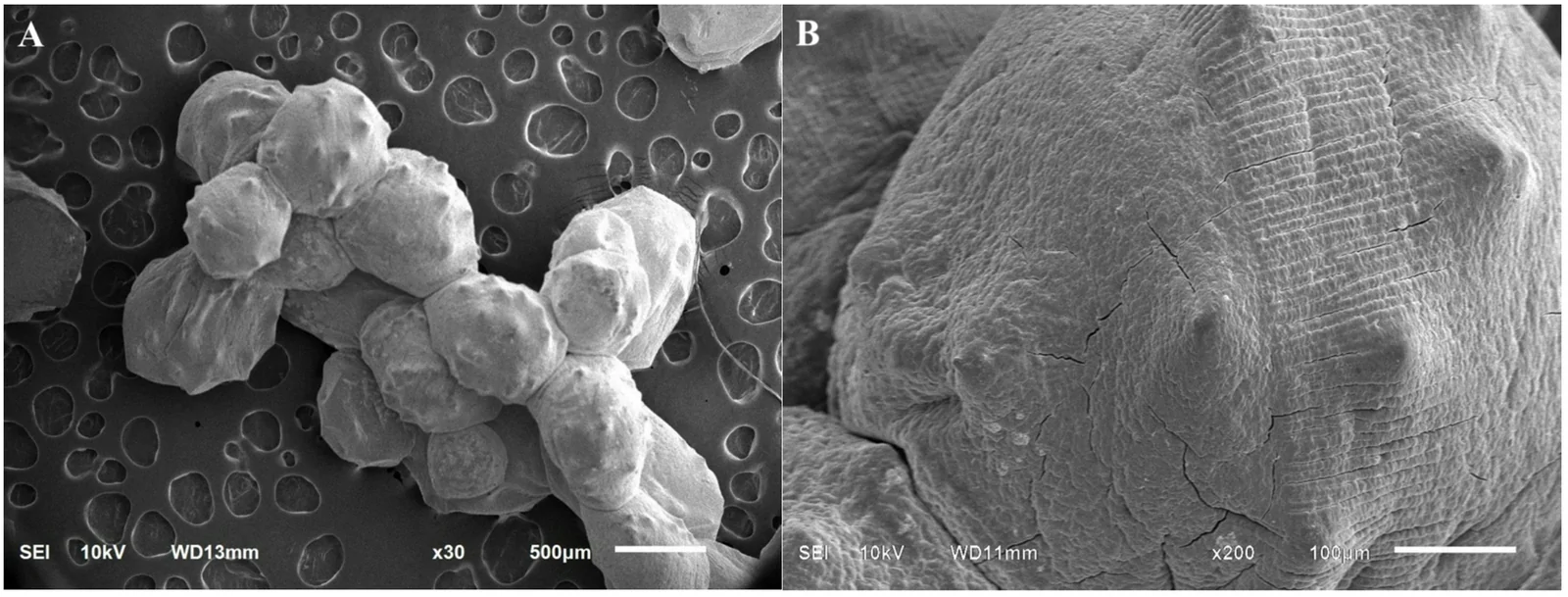
SEM micrograph of LPE microbeads at (**A**) 30 x magnification, (**B**) 200 x magnification.

The particle size and distribution of LPE microbeads were characterized using PSA as described by Kaur et al.^35^. The analysis revealed that the average particle size of the LPE microbeads was 1.2 ± 0.72 μm, with a non-homogeneous distribution, as indicated by a mean/median ratio of 1.462. Morphological observations using SEM at 30× magnification (Fig. 1) showed that the LPE microbeads were predominantly spherical in shape, though not perfectly symmetrical, with a rough and uneven surface. Some microbeads appeared to clump together or form aggregates, likely due to intermolecular forces or residual moisture trapped after the drying process.

**Fig. 2:**
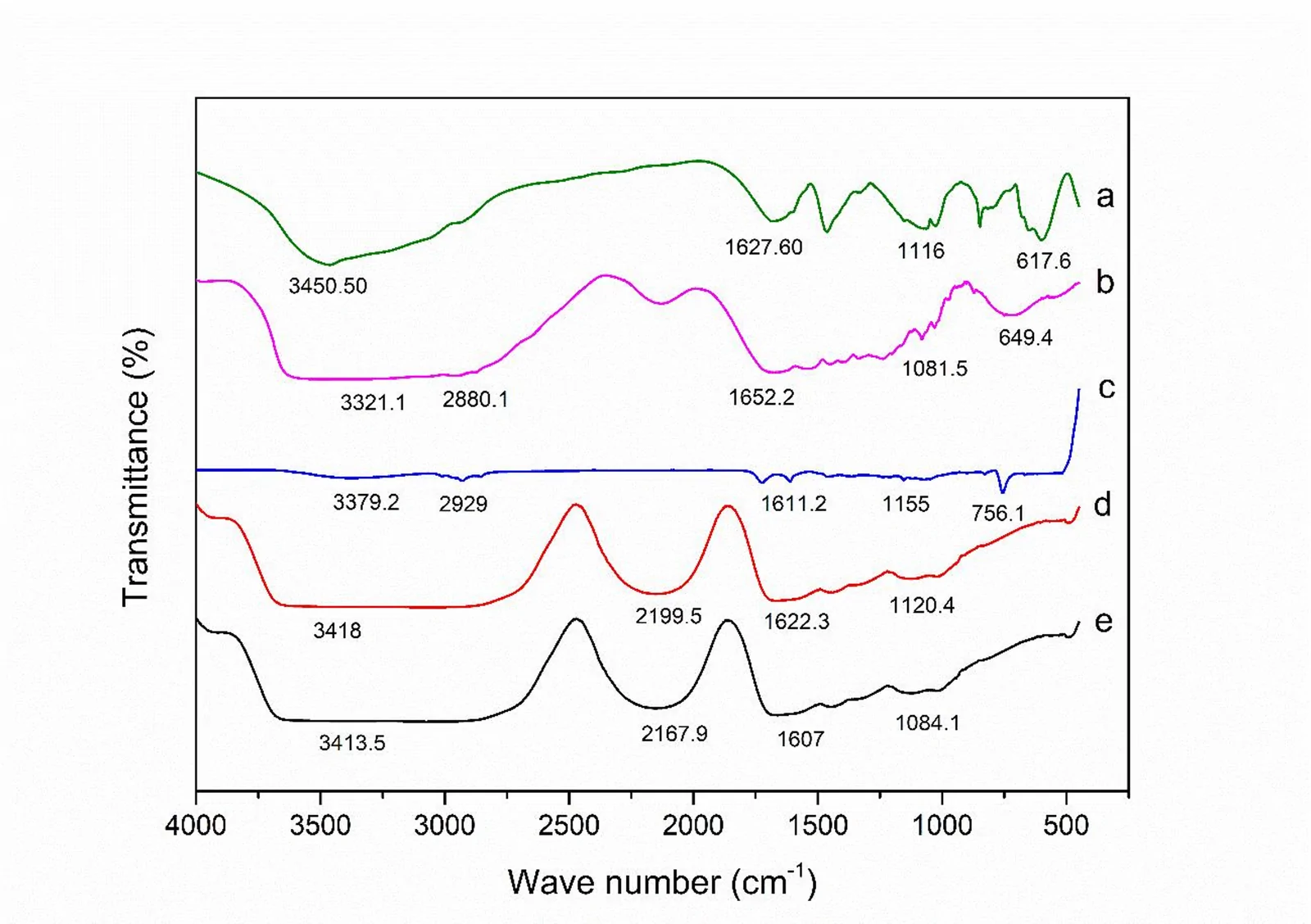
Spectra of FTIR of (a) sodium alginate, (b) gelatin, (c) LPE, (d) coreless microbeads, and (e) LPE microbeads.

Further examination using FTIR spectroscopy was carried out on the microbeads to identify functional groups and confirm the characteristic interactions between the matrix materials and the LPE core (Fig. 2). The FTIR spectrum of sodium alginate (Fig. 2A) exhibited a prominent peak at 3,451 cm⁻¹, corresponding to the characteristic O–H stretching vibration of hydroxyl groups, which is typical of natural polysaccharides. Additional peaks were observed at 1,628 and 1,417 cm⁻¹, representing the asymmetric and symmetric stretching of carboxylate anions (C = O), while the absorption band at 1,116 cm⁻¹ corresponded to C–O ether stretching.

The FTIR spectrum of gelatin (Fig. 2B) displayed a signal at 3,321 cm⁻¹, characteristic of N–H stretching from amine groups. The peak at 2,880 cm⁻¹ indicated aliphatic C–H (sp³) stretching, whereas the bands at 1,652 cm⁻¹ and 1,539 cm⁻¹ were attributed to amide I (C = O and C–N stretching) and amide II (N–H bending) vibrations, respectively. Other peaks were detected in the fingerprint region, including those at 1,451–1,401 cm⁻¹ (C–H and O–H bending) and 1,082 cm⁻¹, corresponding to C–N stretching of amine groups.

Although LPE comprises a mixture of various compounds, its FTIR spectrum (Fig. 2C) revealed dominant functional groups that reflect the main characteristics of the extract. A broad absorption band at 3,379 cm⁻¹ indicated the presence of hydroxyl (–OH) groups, while the peak at 2,929 cm⁻¹ corresponded to aliphatic C–H stretching. The absorption at 1,723 cm⁻¹ was associated with carbonyl (C = O) groups from esters or aldehydes, and the band at 1,611 cm⁻¹ suggested the presence of carbon–carbon double bonds (C = C) in aromatic systems. Additionally, a band at 1,155 cm⁻¹ corresponded to C–O stretching vibrations, likely originating from ether or secondary alcohol groups. Overall, the spectrum supports the presence of phenolic and flavonoid compounds, which are the characteristic bioactive constituents of LPE.

Figure 2D presents the FTIR spectrum of blank microbeads, while Fig. 2E shows that of LPE-loaded microbeads. Both spectra exhibited characteristic amide absorption bands at 3,417 cm⁻¹ and 1,622 cm⁻¹, indicating interactions between the carboxylate groups (COO⁻) of alginate and the amine groups (NH₃⁺) of gelatin, resulting in amide bond formation. Additional peaks were observed at 2,199 cm⁻¹ (C–H sp³ stretching), 1,428 cm⁻¹ (C = O stretching), and in the fingerprint region at 1,309 cm⁻¹ and 1,120 cm⁻¹ (C–H bending), as well as 1,033 cm⁻¹ (C–O stretching).

The FTIR spectrum of the LPE microbeads showed no significant differences compared to the blank microbeads, suggesting the absence of strong chemical interactions between the compounds in LPE and the encapsulating matrix. Thus, it can be concluded that the active compounds in LPE are physically dispersed within the matrix rather than covalently bound, allowing efficient release of the encapsulated substances during application.

**Fig. 3:**
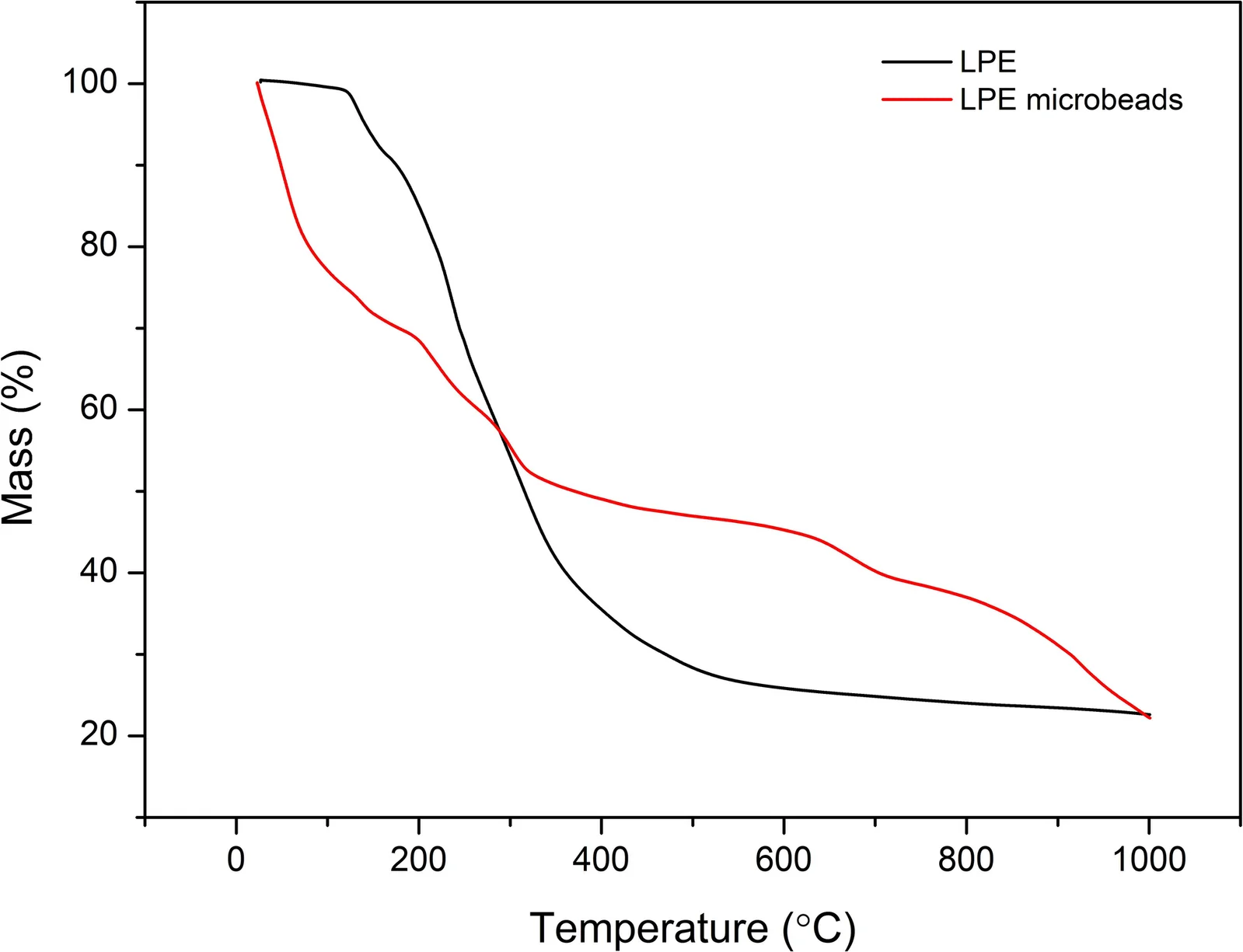
TGA thermograph of LPE and LPE microbeads.

The thermal stability of the LPE microbeads was subsequently evaluated over a temperature range of 26 to 1,000 °C as a representative sample. The TGA thermograms of LPE and LPE microbeads are presented in Fig. 3.

The thermogram (Fig. 3) shows that LPE undergoes mass changes in three distinct stages: below 250 °C, between 250 and 500 °C, and above 500 °C. This pattern indicates that LPE is heat-sensitive due to the presence of thermolabile bioactive compounds. In contrast, the LPE microbeads exhibited a mass loss of 28.21% at temperatures below 200 °C, which can be attributed to the evaporation of volatile components with boiling points lower than 200 °C. A further 49.93% mass reduction was observed between 200 and 500 °C, likely caused by the volatilization of high-boiling-point constituents of LPE.

At temperatures above 500 °C, a sharp decline in mass occurred, corresponding to the thermal decomposition of the polymer matrix involving dehydration and depolymerization processes, accompanied by the cleavage of C–O and C–C bonds and the formation of CO, CO₂, and H₂O^30,33^. The thermogram data indicate that the LPE microbeads exhibit higher thermal stability due to the presence of cross-linking bonds, which help minimize the release of small molecules such as CO₂ and CO. Consequently, the cross-linked matrix provides enhanced thermal resistance and effectively protects the encapsulated LPE from heat-induced degradation.

### The kinetics of LPE release from microbeads

**Fig. 4:**
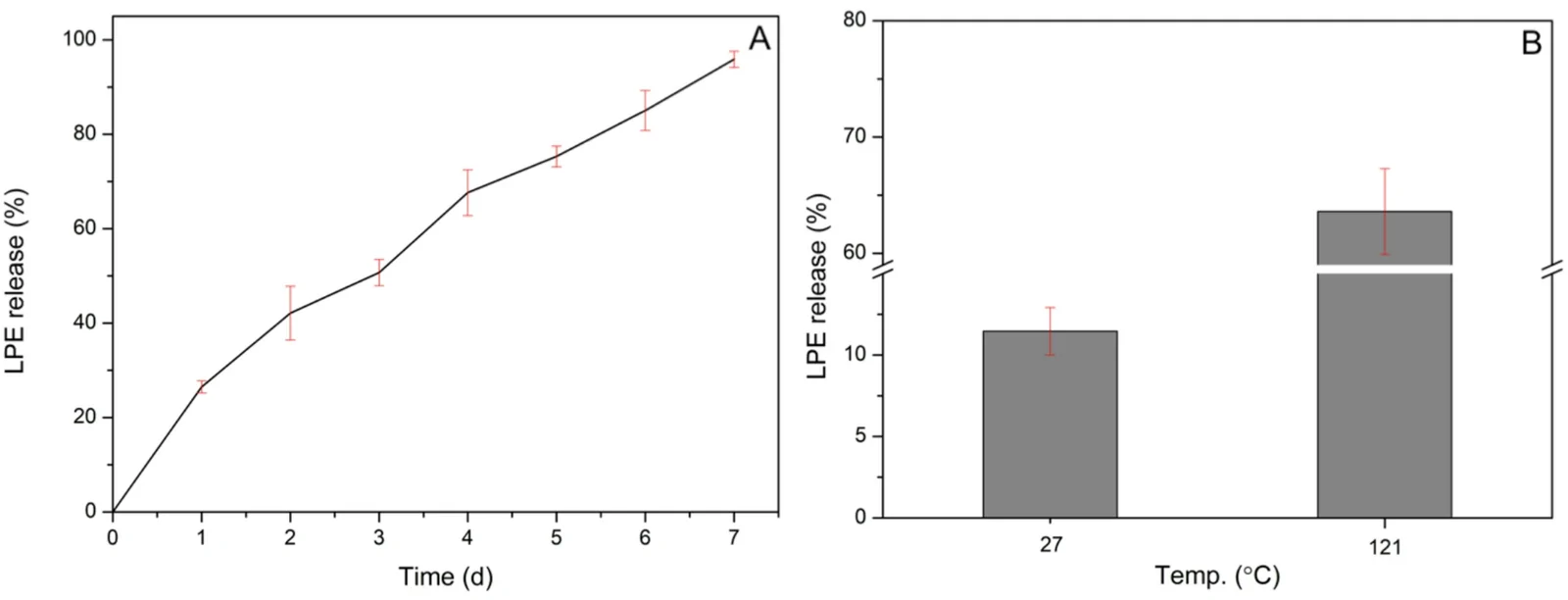
Release rate of LPE from microbeads, (**A**) at room temperature over 7 days and (**B**) under autoclave conditions for 15 min.

Release rate analysis was conducted to determine the amount of LPE released from the microbeads over a specific period. In this study, ethanol was used as the release medium to simulate the conditions of plant tissue culture media. The released LPE was quantified daily over a 7-day period by measuring its absorbance using a UV–Vis spectrophotometer. The obtained absorbance values were compared with the LPE standard calibration curve, and the release profile is presented in Fig. 4.

Figure 4A shows that over seven days, an average of 96% of LPE was gradually released. This gradual release is likely due to the presence of stable amide covalent bonds formed between matrix components, particularly between the carboxylate groups (–COO⁻) of guluronic and mannuronic acid residues in alginate and the amine groups (–NH₂) of gelatin^36^. Such interactions strengthen the microbead structure, resulting in a controlled release system.

In addition, the release test was carried out under two temperature conditions: room temperature (27 °C) and autoclave temperature (121 °C) for 15 min, as shown in Fig. 4B. At room temperature, only 11.46% of LPE was released, indicating the stability of the microbeads at lower temperatures^37^. Conversely, at autoclave temperature, the release increased sharply to 63.61%. This rise was attributed to the weakening of the microbead wall structure under high-temperature exposure, which facilitated the release of active compounds from the bead core^38–40^.

**Fig. 5:**
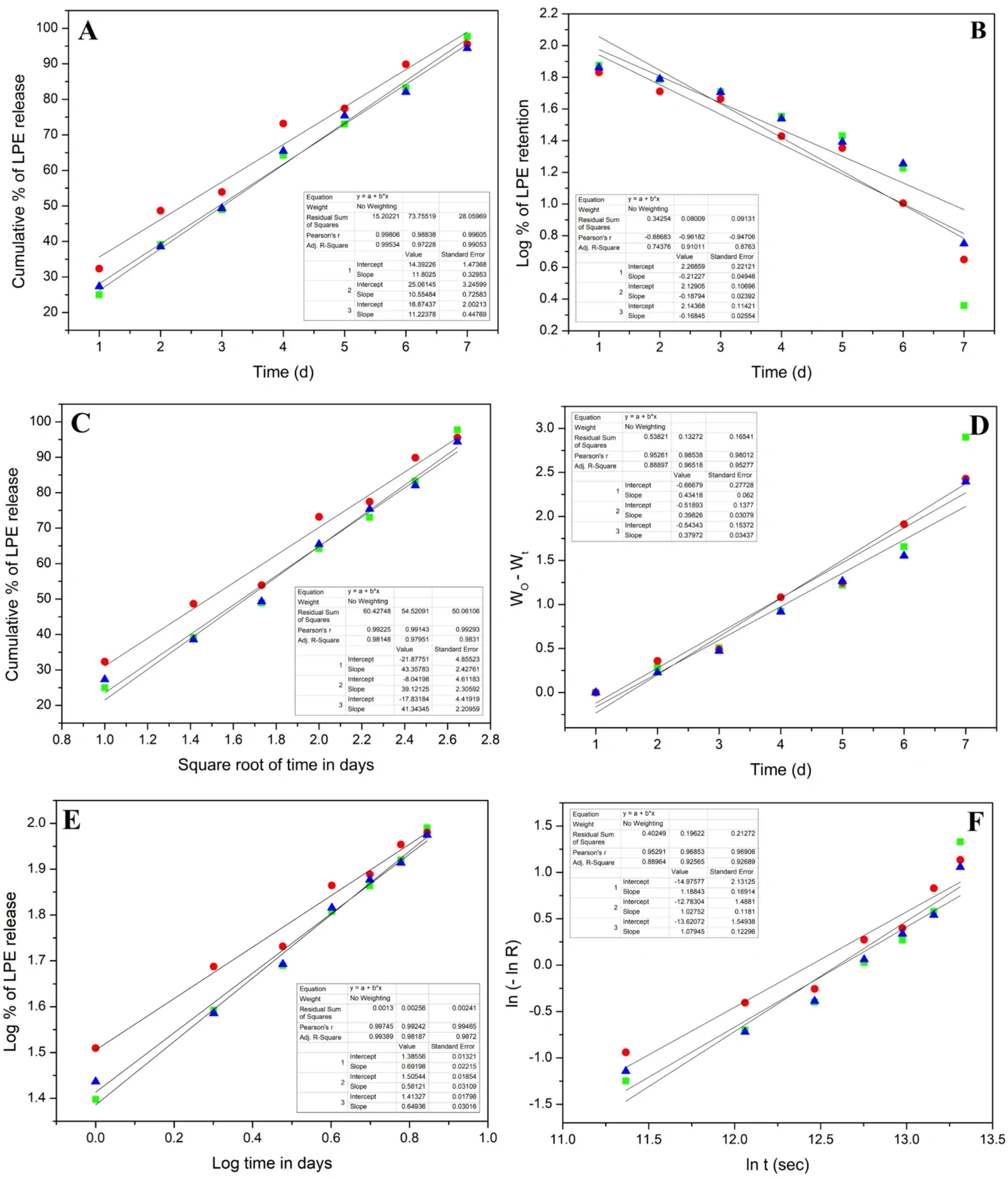
Graph of kinetic models (**A**) zero-order, (**B**) first-order, (**C**) Higuchi, (**D**) Hixon-Crowell, (**E**) Korsmeyer-Peppas, and (**f**) Avrami.

To gain a deeper understanding of the LPE release mechanism, the data were fitted to several kinetic models: zero-order, first-order, Higuchi, Hixson-Crowell, Korsmeyer-Peppas, and Avrami. The goodness-of-fit results for these models are presented in Fig. 5; Table 1.

The Korsmeyer–Peppas model showed the best fit, with a determination coefficient (R²) of 0.9893, indicating high accuracy in describing the release pattern^32^. The diffusion exponent (n) value of 0.6342 suggests that the release mechanism follows a non-Fickian (anomalous transport) model, which involves a combination of diffusion and polymer matrix erosion. This indicates that the release rate is proportional to the remaining amount of LPE within the beads and occurs gradually and in a controlled manner.

**Table 1:** Kinetic models and mechanisms of LPE release from microbeads.

Kinetic models		Repetition			Average
		1	2	3	
Zero-Order	*R* ^*2*^	0.9961	0.9769	0.9921	0.9884
	*k*_*0*_	1.0 × 10^− 4^	1.0 × 10^− 4^	1.0 × 10^− 4^	1.0 × 10^− 4^
First-Order	*R* ^*2*^	0.7865	0.9251	0.8969	0.8695
	*k*_*1*_	2.0 × 10^− 6^	2.0 × 10^− 6^	2.0 × 10^− 6^	2.0 × 10^− 6^
Higuchi	*R* ^*2*^	0.9848	0.9829	0.9859	0.9845
	*k*_*hc*_	0.1475	0.1331	0.1407	0.1404
Hixson-Crowell	*R* ^*2*^	0.9075	0.9710	0.9606	0.9464
	*k*_*H*_	5.0 × 10^− 6^	5.0 × 10^− 6^	4.0 × 10^− 6^	4.7 × 10^− 6^
Korsmeyer-Peppas	*R* ^*2*^	0.9949	0.9849	0.9893	0.9893
	*k*_*K*_	0.0093	0.0543	0.0161	0.0266
	*n*	0.6920	0.5612	0.6494	0.6342
Avrami	*R* ^*2*^	0.9080	0.9380	0.9391	0.9284
	*k*_*A*_	3.37 × 10^− 6^	3.95 × 10^− 6^	3.31 × 10^− 6^	3.54 × 10^− 6^
	*n*	1.1884	1.0275	1.0795	1.0985

Furthermore, based on the release rate constant of 0.0266 s⁻¹, the presence of two polymer matrix layers (alginate and gelatin) was found to significantly slow down the release of LPE. This is supported by strong ionic interactions between the carboxylate groups of alginate and the amino groups of gelatin, as well as structural reinforcement by CaCl₂ as a cross-linking agent^41^. Therefore, these microbeads effectively function as a stable and sustained delivery system for active compounds in plant tissue culture applications.

### Microcapsule storage stability

**Fig. 6:**
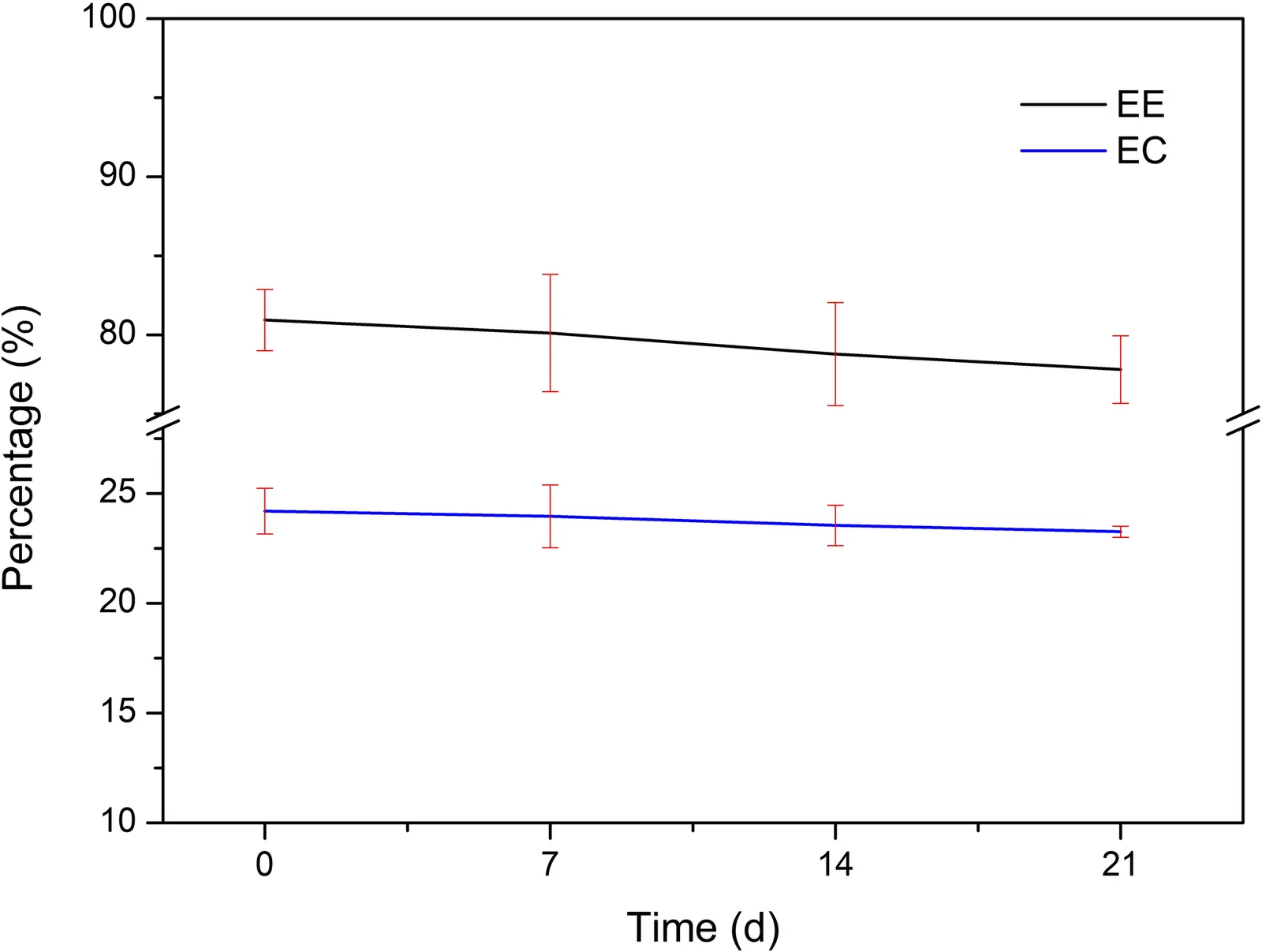
Stability of LPE microbeads at room temperature.

The storage stability of LPE microbeads was evaluated under room temperature conditions, as shown in Fig. 6. During three weeks of storage, the EE decreased to 77.8 ± 2.14%, and the EC dropped to 23.26 ± 0.25%. This decline was attributed to the gradual release of LPE during the storage period, which is associated with physical and chemical changes in the coating layer and the diffusion of active compounds from within and on the surface of the microbeads. Over time, the coating layer tends to absorb oxygen from the environment, which then diffuses into the bead structure and triggers the formation of peroxide compounds inside^42^. Previous studies have also shown that the stability of encapsulated active ingredients is strongly influenced by several factors, including the structure and properties of the coating matrix, processing conditions, and interactions between the encapsulating materials and the core compounds^43^.

### Browning measurement

To evaluate the effect of LPE and its microbeads on browning in *Musa* spp. explants, observations were made on the browning index during the initiation stage of Kepok Tanjung and Barangan banana explants (Table 2).

**Table 2:** Browning index of Kepok Tanjung and Barangan explants.

Treatments	Concentration (mg/mL)	Browning index (%)	
		Kepok Tanjung	Barangan
A	0	72.22^a^	83.33^a^
LPE **1**	0.66	61.11^b, A,*^	69.44^b, A,*^
LPE **2**	1.32	47.22^cd, A,**^	61.11^bc, A,*^
LPE **3**	1.98	47.22^cd, A,**^	55.56^cd, A,**^
*Microbeads* **1**	2.73	52.78^bc, B,*^	55.56^cd, B,*^
*Microbeads* **2**	5.46	44.44^cd, B,**^	47.22^de, B,*^
*Microbeads* **3**	8.19	38.89^d, B,**^	41.67^e, B,**^
*P-value*			
Treatment		< 0.05	< 0.05
Encapsulation		< 0.05	< 0.05
Concentration		< 0.05	< 0.05
Encapsulation × concentration		0.530	0.757

Mean values in the same column followed by different lowercase superscript letters indicate significant differences due to treatment interactions; different uppercase letters indicate significant differences between encapsulated and non-encapsulated samples; and different symbols indicate significant differences among concentrations.

Table 2 shows that both encapsulation and concentration factors significantly affected the browning index of Kepok Tanjung and Barangan banana explants. The best results were obtained with the Microbeads 3 treatment, which reduced the browning index by 38.89% in Kepok Tanjung and 41.67% in Barangan explants. This indicates that LPE in microbead form was more effective in suppressing browning than unencapsulated LPE.

Browning in explants during the early initiation stage is generally triggered by mechanical wounding during explant excision, which leads to the disruption of cellular compartmentalization. This damage causes the release of phenolic compounds from the vacuole and increases their interaction with oxidative enzymes such as polyphenol oxidase (PPO) and peroxidase (POD)^7^. In addition, wounding stress induces the formation of reactive oxygen species (ROS), which further accelerates the oxidation of phenolic compounds into quinones that are toxic and brown in color^8^. The accumulation of these compounds not only results in tissue browning but also reduces explant viability and inhibits growth during the early stage of in vitro culture.

In this context, the application of LPE, which is rich in antioxidant compounds, becomes highly relevant for reducing browning. LPE has been reported to contain major bioactive compounds such as nobiletin, tangeretin, sinensetin, tetramethylscutellarein, and scoparone, which belong to the polymethoxyflavone group, as well as chlorophyll derivatives such as pyropheophorbide A^26^. These polymethoxyflavones have been widely reported as dominant constituents in *Citrus* peels^44–47^ and are known to exhibit strong antioxidant activity by donating electrons to neutralize free radicals, as well as inhibiting PPO and POD activity^48–52^. Meanwhile, the presence of pyropheophorbide A indicates the contribution of chlorophyll-derived compounds involved in pigment metabolism^53,54^. The variability in chemical composition reflects the complexity of plant extracts, which is influenced by genetic and environmental factors, thereby enabling potential synergistic effects in browning inhibition.

However, the effectiveness of LPE is strongly influenced by the stability and availability of its active compounds during culture conditions. Therefore, encapsulation within an alginate–gelatin matrix plays a crucial role in protecting bioactive compounds from degradation while regulating their release. This is consistent with the present findings, where LPE microbeads resulted in a greater reduction in browning index compared to non-encapsulated LPE, with the Microbeads **3** treatment showing the lowest values in both explant types.

**Fig. 7:**
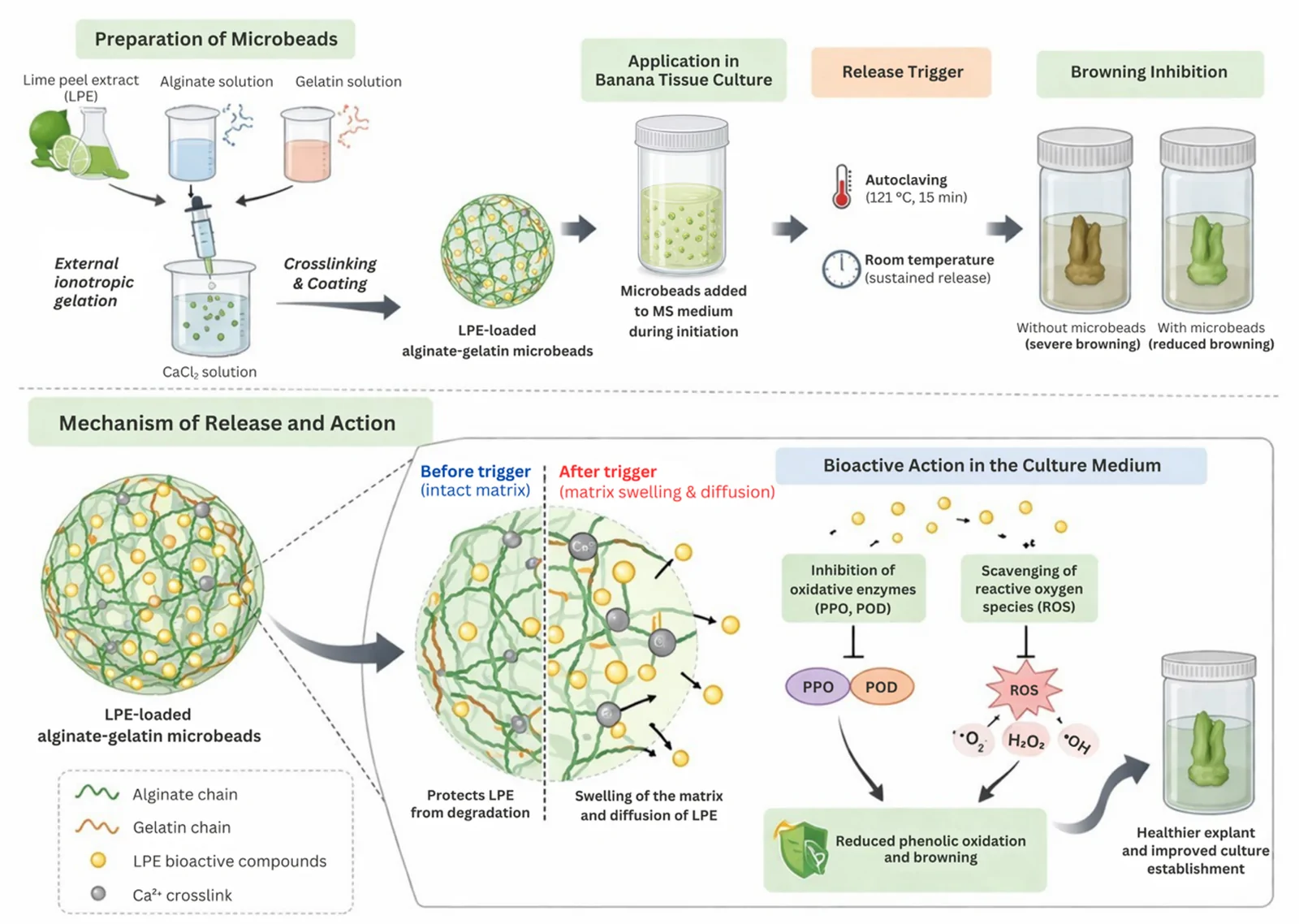
Schematic illustration of (top) the preparation of LPE-loaded alginate-gelatin microbeads, their application in banana tissue culture, and (bottom) the proposed release mechanism and mode of action.

This mechanism is further supported by the release study, in which the microbeads exhibited minimal release at room temperature, indicating good stability under initial conditions. In contrast, exposure to autoclave temperature significantly increased the release to 63.61% due to the weakening of the microbead matrix structure. These results suggest that the sterilization process not only ensures aseptic conditions but also acts as a trigger for the initial burst release of bioactive compounds. Consequently, after sterilization, active compounds are readily available to inhibit enzymatic browning during the early stage of culture. Furthermore, the sustained release of LPE from the microbeads during the initiation phase ensures prolonged antioxidant activity, thereby maintaining tissue stability and improving explant viability. A schematic illustration of the release mechanism during and after autoclave treatment is presented in Fig. 7.

Overall, the combination of a bioactive-rich extract and a microbead-based delivery system provides a dual mechanism, namely protection against degradation and controlled release of active compounds. This synergistic effect contributes to more effective browning suppression compared to free extract application, highlighting the potential of this system for plant tissue culture applications. However, it should be noted that the encapsulation process introduces additional formulation steps that may influence process efficiency under practical conditions. In addition, the scalability of microbead production and consistency of release behavior still require further evaluation. Variability in extract composition, influenced by raw material sources and environmental factors, may also affect reproducibility. Therefore, future studies should focus on process optimization, standardization of extract composition, and validation under larger-scale culture systems to support broader application.

## Conclusion

The LPE microbeads obtained in this study exhibited good encapsulation performance, with stable morphology, satisfactory storage and thermal stability, and a controlled release pattern consistent with the Korsmeyer–Peppas kinetic model. Their application effectively reduced browning in Kepok Tanjung and Barangan explants, demonstrating the advantage of encapsulated LPE over its free form. These results suggest that LPE microbeads hold strong potential as a controlled delivery system in plant tissue culture. Future studies may explore their broader use in other plant species or for encapsulating different bioactive compounds to enhance growth and stress tolerance.

## Data Availability

All data generated or analysed during this study are included in this published article.
